# Metabolic syndrome in Australia: nationwide survey results by remoteness and Indigenous status, 2012–2019

**DOI:** 10.1038/s41366-025-02013-y

**Published:** 2026-01-08

**Authors:** Utpal K. Mondal, M. Mamun Huda, Anayochukwu E. Anyasodor, Sok Cheon Pak, Bernd H. Kalinna, Feleke H. Astawesegn, Subash Thapa, Kedir Y. Ahmed, Setognal B. Aychiluhm, Shakeel Mahmood, Md. Ferdous Rahman, Muhammad J. A. Shiddiky, Allen G. Ross

**Affiliations:** 1 Rural Health Research Institute, Charles Sturt University, Orange, NSW Australia; 2 School of Rural Medicine, Charles Sturt University, Orange, NSW Australia; 3 School of Dentistry and Medical Sciences, Charles Sturt University, Bathurst, NSW Australia; 4 College of Medicine, Ajman University, Ajman, United Arab Emirates

**Keywords:** Risk factors, Epidemiology

## Abstract

**Background:**

In Australia, the rising prevalence of metabolic syndrome (MetS) presents a significant public health challenge. However, research on geographic and ethnic disparities remains limited. This study aimed to investigate the prevalence and temporal trends of MetS by geographic remoteness and between Indigenous and non-Indigenous Australians.

**Methods:**

We analysed data from 44,760 adults (aged ≥18 years) derived from the National Health Survey (2014–2015 and 2017–2018) and the National Aboriginal and Torres Strait Islander Health Survey (2012–2013 and 2018–2019). Weighted prevalence estimates of MetS were calculated overall and stratified by remoteness. The Average Annual Rate of Change (AARC) in MetS prevalence was computed to assess temporal trends.

**Results:**

MetS prevalence varied notably by remoteness and ethnicity. In the most recent surveys, 7.1% (95% CI: 6.19–8.19) of Indigenous adults (2018–2019) and 4.6% (95% CI: 4.23–4.99) of non-Indigenous adults (2017–2018) had MetS. Prevalence was higher in remote areas for both groups. Among non-Indigenous adults, MetS declined across most regions but increased in remote areas from 4.5% to 7.1% (AARC: +15.77%), while among Indigenous adults it remained stable in remote areas but rose in major cities and regional settings. Central obesity and type 2 diabetes (T2D) were the most prominent contributors to MetS among Indigenous adults, whereas hypertension and high cholesterol were more prevalent among non-Indigenous adults in regional areas. Central obesity was the most common MetS risk factor, affecting 57.4% (95% CI: 55.14–59.63) of Indigenous and 40.9% (95% CI: 39.90–41.85) of non-Indigenous adults. High cholesterol was the least common risk factor among Indigenous adults (7.6% [95% CI: 6.58–8.67]), whereas elevated blood sugar was the least common among non-Indigenous adults (4.8% [95% CI: 4.44–5.21]).

**Conclusions:**

Substantial disparities in MetS exist across Australia, disproportionately affecting Indigenous Australians and residents of remote areas. Culturally tailored, region-specific interventions targeting obesity are urgently needed through Local Health Districts and Aboriginal Community Controlled Health Organisations.

## Introduction

Non-communicable diseases (NCDs) are the leading cause of death and disability worldwide, responsible for approximately 43 million deaths annually and accounting for 75% of global mortality [1]. Their rising prevalence places substantial pressure on health care systems, economic productivity, and overall societal well-being [2–5]. Metabolic syndrome (MetS), a clustering of interrelated metabolic abnormalities, is increasingly recognised as a global public health threat [6–9]. It is characterised by the presence of at least three out of five cardiometabolic risk factors: abdominal (central) obesity, elevated blood pressure, hyperglycaemia, hypertriglyceridemia, and reduced high-density lipoprotein (HDL) cholesterol levels [6, 10, 11]. Collectively, these factors markedly accelerate the risk of major NCDs, including cardiovascular disease (CVD), type 2 diabetes (T2D), stroke, macular degeneration, and chronic kidney disease (CKD) [12–15]. Globally, MetS affects approximately one-quarter of adults, and its prevalence is rising in many regions, including Australia [8, 9].

In Australia, the increasing prevalence of MetS is influenced by a complex interplay of socioeconomic, demographic, and lifestyle factors [11, 16, 17]. Recent evidence highlights significant rural–urban disparities in MetS prevalence, with rural and remote areas often disproportionately affected [18–20]. These disparities in MetS prevalence are compounded by broader social determinants of health, including limited access to health care services, economic disadvantage, and lifestyle factors associated with rural living (e.g., lower physical activity and higher obesity prevalence) [18]. Australians living in rural and remote areas experience a higher burden of chronic conditions, including obesity, hypertension, and T2D than their urban counterparts, as documented by the Australian Institute of Health and Welfare (AIHW) [18]. Aboriginal and Torres Strait Islander peoples (hereafter respectfully referred to as Indigenous) face an even greater burden as they are 3.3 times more likely to have diabetes than non-Indigenous Australians [18–21], and tend to develop metabolic risk factors at a younger age (teenage years) [22]. The intersection of ethnicity and remoteness further amplifies health disparities, particularly in remote Indigenous communities. Despite the clinical and public health importance of MetS as a clustering of risk factors, research on its prevalence and geographic distribution in Australia remains insufficient.

Similar patterns have been reported among Indigenous populations in other nations with colonial histories. In Canada, studies among Cree, First Nations, and Métis adults indicate MetS prevalence frequently exceeding 40%, substantially higher than non-Indigenous Canadians [23–25]. In New Zealand, population-based surveys and regional cohort studies show Māori and Pacific adults have nearly double the prevalence of MetS compared with people of European descent, with central obesity and dyslipidaemia being key contributors [26–28]. In the United States, American Indian and Alaska Native populations, including participants in the Pima and Strong Heart cohorts, record some of the highest adult prevalence worldwide, approaching 60% in certain groups and well above rates observed the general population [29, 30].

The National Health Survey (NHS) and the National Aboriginal and Torres Strait Islander Health Survey (NATSIHS), both conducted by the Australian Bureau of Statistics (ABS), provide nationally representative data that enable the assessment of MetS prevalence across diverse population groups and geographic regions in Australia [31, 32]. Although MetS is increasingly recognised as a major public health concern, critical knowledge gaps remain regarding its temporal trends and geographic variation, particularly in rural and remote settings. Most existing studies have focused on urban populations or specific regions, often overlooking the unique health challenges faced by people living outside major cities [33–36]. To our knowledge, this is the first comprehensive study to examine MetS prevalence and trends across all levels of geographic remoteness in Australia, using nationally representative samples of both Indigenous and non-Indigenous adults. The study aimed to estimate the prevalence of MetS among Indigenous and non-Indigenous Australians across urban, regional and remote areas, and to analyse temporal trends between 2012 and 2019. These findings are intended to provide evidence-based insights to inform public health policies, guide equitable resource allocation, and ultimately reduce the burden of MetS in underserved rural and Indigenous communities across Australia.

## Methods

### Data sources

We conducted a secondary analysis of data drawn from two nationally representative population health surveys administered by the ABS: the NHS and the NATSIHS. The NHS is a periodic cross-sectional survey of the general Australian population’s health status and health-related behaviours, while the NATSIHS specifically surveys the health of Aboriginal and Torres Strait Islander population. For this study, we included two waves of each survey that contained relevant metabolic health data: the 2014–2015 and 2017–2018 NHS, and the 2012–2013 and 2018–2019 NATSIHS. Although the ABS released the 2022–2023 NHS and NATSIHS datasets during our study period, confidential unit record files were not accessible through the ABS DataLab at the time of analysis. Given this restriction and project timelines, we proceeded with the most recent available datasets. Together, these surveys provide a robust, population-wide profile of MetS prevalence and associated risk factors among both Indigenous and non-Indigenous Australian adults.

### Sampling design

Both the NHS and NATSIHS employed stratified multistage random sampling methods to ensure national and regional representativeness. In the NHS, private dwellings were randomly selected, and within each household one adult ( ≥ 18 years) and one child (0–17 years) were randomly invited to participate. Proxy interviews were permitted for adults who were unable to respond due to illness, disability, or language barriers. The NATSIHS used a comparable multistage sampling strategy, specifically designed to reflect the diversity of the Aboriginal and Torres Strait Islander communities. The sample comprised two components: a community sample (from discrete Indigenous communities) and a non-community sample (from private dwellings). Operational exclusions were applied to some areas (e.g., Tasmania, and certain non-remote parts of Western Australia and the Northern Territory) to maximise feasibility and sample quality. To ensure geographic representativeness, targeted sampling was conducted in the Torres Strait region. In non-remote households, up to two adults and two children were randomly selected, while in remote households, up to one adult and one child were included per dwelling.

### Data collection

Data were collected through structured face-to-face interviews by trained ABS interviewers. The NHS and NATSIHS recorded demographic and socioeconomic characteristics; smoking and alcohol consumption; diet and physical activity; health care access; and self-reported long-term conditions. Where available, standardised physical measurements (height, weight, waist circumference, and blood pressure) were obtained. The NATSIHS additionally included culturally relevant questions to reflect the unique social and environmental contexts of Indigenous communities. Health conditions in both surveys were classified according to the International Classification of Diseases, 10th Revision (ICD-10) to identify the individual components of MetS [37]. Missing physical measurements (height, weight, waist circumference, and blood pressure) were imputed by the ABS using hot-deck donor methods based on respondent characteristics. We undertook no additional imputation, and no laboratory biomarkers were imputed [32, 38].

### Selection of the analytic sample

We restricted the analysis to adults aged 18 years and older. Across both waves of the NHS, a total of 40,572 individuals were surveyed. After excluding children (*n* = 9642) and Indigenous participants (*n* = 750) to avoid duplication with the NATSIHS dataset, 30,180 non-Indigenous adults were retained for analysis. Similarly, the NATSIHS datasets included 23,526 individuals, and following the exclusion of children (*n* = 8946), 14,580 Indigenous adults remained eligible for analysis. The final analytic sample comprised 44,760 adults, including 30,180 non-Indigenous and 14,580 Indigenous individuals.

### Outcome measurements

The primary outcome of this study was the presence of MetS, defined using criteria adapted from internationally recognised guidelines, including the National Cholesterol Education Program Adult Treatment Panel III (NCEP ATP III) and the International Diabetes Federation (IDF) [7, 10, 39]. However, due to limitations in lipid biomarker (HDL Cholesterol and triglycerides) availability across survey cycles, in this study MetS was defined using only four of the five interrelated cardiometabolic risk factors: (1) central obesity, defined as a waist circumference of ≥94 cm for males and ≥80 cm for females; (2) T2D, defined as meeting any of the following criteria: a self-reported diagnosis of diabetes (excluding gestational diabetes), self-reported elevated blood glucose levels previously diagnosed by a health professional, or current use of glucose-lowering medication, consistent with ICD-10 code E11 [40]; (3) hypertension, defined as a self-reported diagnosis of hypertension, measured systolic blood pressure ≥140 mmHg and/or diastolic blood pressure ≥90 mmHg, or current use of antihypertensive medication; and (4) high cholesterol, defined as a self-reported diagnosis of hyperlipidaemia (ICD-10 code E78) [40] or current use of lipid-lowering medication.

### Exposure of interest

The primary exposure variable in this study was geographic remoteness, defined according to the Australian Statistical Geography Standard (ASGS) Remoteness Structure developed by the ABS [41]. The ASGS categorises areas into five standard levels of remoteness: Major Cities, Inner Regional, Outer Regional, Remote, and Very Remote. To ensure sufficient statistical power across strata, and in line with ABS reporting practices, the Remote and Very Remote categories were combined into a single group. Accordingly, the final exposure variable included four categories: Major Cities, Inner Regional, Outer Regional, and Remote. This variable was used to examine spatial variation in the prevalence of MetS across different levels of service accessibility and population distribution.

### Statistical analysis

All statistical analyses were conducted using Stata version 18.0 [42]. Survey weights provided by the ABS were applied to account for the complex survey design, differential selection probabilities, and non-response. Weighted analyses were conducted separately for the NHS and NATSIHS to generate nationally representative estimates of MetS prevalence among non-Indigenous and Indigenous populations, respectively. Both the NHS and NATSIHS employed multistage, stratified sampling designs, and provided corresponding survey weights. These weights were incorporated to adjust for unequal sampling probabilities and participant non-response, ensuring that prevalence estimates accurately reflected the national population distributions [38]. Population-weighted estimates of MetS prevalence were calculated for each survey year, stratified by Indigenous status and geographic remoteness. To assess temporal trends, the AARC in MetS prevalence was estimated between the earliest and most recent survey cycles for each subgroup. The AARC was calculated using a method adapted from the United Nations Children’s Fund (UNICEF) approach [43], which assumes a constant annual percentage change over time. According to this method, the prevalence is denoted as Yt and the AARC remains constant at *b*%, the prevalence of *n*th year (Yt + *n*) can thus be calculated:

$$\mathrm{Yt}+n={\mathrm{Yt}}^{* }{\left(1-b \% \right)}^{{\rm{n}}}$$

With the estimated slope *β*, we computed average annual rate of reduction (AARR) = 1 − exp (*β*), and average annual rate of increment (AARI) as AARI = exp (*β*) − 1. Therefore, an AARC with a negative value was reported as a reduction, while a positive value was regarded as an increment.

### Ethics

This study was approved by the Charles Sturt University Human Research Ethics Committee (Protocol number #2023-118). The NHS and NATSIHS surveys were conducted with informed consent and under ethical oversight. De-identified data were accessed through the secure ABS DataLab. All analyses adhered to the Strengthening the Reporting of Observational Studies in Epidemiology (STROBE) guideline for cross-sectional studies [44].

## Results

### Characteristics of study participants

A total of 44,760 adults were included in the study, comprising 30,180 non-Indigenous and 14,580 Indigenous participants. Overall, non-Indigenous adults represented the majority (60%) of the combined study population. Both groups showed comparable age and sex distributions, with older adults ( ≥ 45 years) comprising the largest proportion in each group and younger adults (18–29 years) the smallest. However, younger adults were more prominently represented among the Indigenous participants (27.1%) compared to the non-Indigenous participants (14.6%). In both populations, females outnumbered males (58.6% of Indigenous and 53.8% of non-Indigenous participants) (Table 1). Geographic distribution of residence varied markedly by Indigenous status: most non-Indigenous participants resided in Major Cities (64.3%), with only 2.6% living in Remote areas. In contrast, Indigenous participants were far more likely to live in Remote areas (41.8%) than in Major Cities (21.1%) (Table 1).

**Table 1:** Sociodemographic characteristics of the study population by survey type and year.

Category	NHS (Non-Indigenous Australians)			NATSIHS (Indigenous Australians)		
Survey year	2017–2018	2014–2015	Total	2018–2019	2012–2013	Total
Survey participants (*N*)	21,315	19,257	40,572	10,579	12,947	23,526
Study participants (*N*)	15,927	14,253	30,180	6423	8157	14,580
Age group (% [*n*])						
18–29 years	13.9 (2207)	15.3 (2187)	14.6 (4394)	25.8 (1660)	28.1 (2289)	27.1 (3949)
30–44 years	26 (4149)	28.1 (4005)	27 (8154)	30.1 (1932)	33.5 (2730)	32.0 (4662)
45+ years	60.1 (9571)	56.6 (8061)	58.4 (17,632)	44.1 (2831)	38.5 (3138)	41.0 (5969)
Sex; % (*n*)						
Male	46.5 (7400)	45.8 (6528)	46.1 (13,928)	41.4 (2662)	41.4 (3375)	41.4 (6037)
Female	53.5 (8527)	54.2 (7725)	53.8 (16,252)	58.6 (3761)	58.6 (4782)	58.6 (8543)
Remoteness (% [*n*])						
Major cities	62.2 (9908)	66.7 (9512)	64.3 (19,420)	26.0 (1668)	17.3 (1415)	21.1 (3083)
Inner regional	20.3 (3230)	18.4 (2620)	19.4 (5850)	18.0 (1154)	8.0 (648)	12.4 (1802)
Outer regional	14.5 (2318)	12.6 (1803)	13.6 (4121)	17.4 (1120)	7.1 (576)	11.6 (1696)
Remote	3.0 (471)	2.2 (318)	2.6 (789)	38.6 (2481)	44.2 (3608)	41.8 (6089)

### MetS prevalence and risk factors in the latest survey period

In the latest survey cycle for each population, the overall prevalence of MetS was higher among Indigenous adults than non-Indigenous adults. MetS affected 7.1% (95% CI: 6.19–8.20) of Indigenous Australians in 2018–2019, compared to 4.6% (95% CI: 4.23–4.99) of non-Indigenous Australians in 2017–2018 (Table 2). Among the individual MetS risk factors, central obesity was the most prevalent component in both groups. In 2018–2019, 57.4% (95% CI: 55.14–59.63) of Indigenous adults had central obesity, compared to 40.9% (95% CI: 39.90–41.85) of non-Indigenous adults in 2017–2018. In contrast, high cholesterol was the least common risk factor among Indigenous adults (7.6%, 95% CI: 6.58–8.67), while elevated blood sugar (T2D) was the least common among non-Indigenous adults (4.8%, 95% CI: 4.44–5.21) (Table 2). Notably, the prevalence of diagnosed elevated blood sugar was more than twice as high in Indigenous adults (9.9%, 95% CI: 8.85–11.06) than in non-Indigenous adults (4.8%, 95% CI: 4.44–5.21). The prevalence of hypertension was similar between groups (13.9% in Indigenous vs. 13.7% in non-Indigenous) (Table 2).

**Table 2:** Prevalence of metabolic syndrome and related conditions across Australian Indigenous and non-Indigenous populations.

Condition	Population group	Previous survey^c^		Latest survey^d^		AARC
		*n*^a^	% (95% CI)^b^	*n*^a^	% (95% CI)^b^	
Metabolic syndrome	Non-Indigenous (NHS)	888	5.5 (5.11, 6.03)	860	4.6 (4.23, 4.99)	−6.07
	Indigenous (NATSIHS)	433	4.1 (3.59, 4.7)	597	7.1 (6.19, 8.19)	9.59
• Central obesity	Non-Indigenous (NHS)	6025	39.9 (38.9, 40.98)	7060	40.9 (39.90, 41.85)	0.78
	Indigenous (NATSIHS)	4103	55.0 (53.35, 56.66)	3973	57.4 (55.14, 59.63)	0.71
• Type 2 diabetes	Non-Indigenous (NHS)	794	5.0 (4.62, 5.52)	929	4.8 (4.44, 5.21)	−1.61
	Indigenous (NATSIHS)	1033	9.8 (8.91, 10.69)	856	9.9 (8.85, 11.06)	0.24
• Hypertension	Non-Indigenous (NHS)	2355	14.6 (13.86, 15.30)	2544	13.7 (13.06, 14.34)	−2.06
	Indigenous (NATSIHS)	1112	9.5 (8.68, 10.41)	1106	13.9 (12.53, 15.34)	6.5
• High cholesterol	Non-Indigenous (NHS)	1475	9.1 (8.58, 9.73)	1466	7.8 (7.36, 8.35)	−4.98
	Indigenous (NATSIHS)	510	4.7 (4.15, 5.35)	615	7.6 (6.58, 8.67)	8.17

*NHS* national health survey, *NATSIHS* National Aboriginal and Torres Strait Islander Health Survey, *CI* confidence interval, *AARC* average annual rate of change (negative and positive sign represents a decrease and an increase in the prevalence, respectively).

^a^Unweighted frequency.

^b^Weighted estimates.

^c^2012–2013 and 2014–2015 for Indigenous and Non-Indigenous survey, respectively.

^d^2018–2019 and 2017–2018 for Indigenous and Non-Indigenous survey, respectively.

### Geographic variations in MetS prevalence and risk factors

MetS prevalence varied significantly by geographic remoteness in both populations. Overall, rural and remote areas had higher MetS prevalence than urban areas for both Indigenous and non-Indigenous Australians (Table 3). Among non-Indigenous adults in 2017–2018, the MetS prevalence ranged from 4.3% (95% CI: 3.9–4.8) in Major Cities to 7.1% (95% CI: 3.9–12.6) in Remote areas. The Indigenous population showed a somewhat different pattern: in 2018–2019 the highest MetS prevalence was observed in Inner Regional areas (8.1%, 95% CI: 6.2–10.4), slightly exceeding the Remote area prevalence (7.8%, 6.5–9.4), while the lowest was in Major Cities (5.9%, 4.5–7.8). Despite minor differences in the rank order of prevalence, both Indigenous and non-Indigenous groups exhibited their lowest MetS prevalence in Major Cities and higher rates in all non-urban regions (Table 3).

**Table 3:** Prevalence and annual average rate of change (AARC) of metabolic syndrome and major risk factors by remoteness and population group in Australia.

Remoteness	Population group	Survey year	Metabolic syndrome (%; 95% CI)^a^	Central obesity (%; 95% CI)^a^	Type 2 diabetes (%; 95% CI)^a^	Hypertension (%; 95% CI)^a^	High cholesterol (%; 95% CI)^a^
Major cities	Non-Indigenous	NHS 2014–2015	5.1 (4.57, 5.64)	37.1 (35.89, 38.3)	4.6 (4.13, 5.15)	13.2 (12.37, 14)	8.8 (8.17, 9.52)
		NHS 2017–2018	4.3 (3.87, 4.75)	38.4 (37.3, 39.59)	4.5 (4.03, 4.93)	12.6 (11.9, 13.38)	7.6 (7.02, 8.19)
		**AARC**	−5.47	1.21	−1.16	−1.39	−4.92
	Indigenous	NATSIHS 2012–2013	2.5 (1.78, 3.66)	52.0 (48.55, 55.41)	8.3 (6.74, 10.12)	7.4 (5.83, 9.39)	3.8 (2.84, 5.16)
		NATSIHS 2018–2019	5.9 (4.52, 7.81)	55.3 (51.02, 59.55)	7.5 (5.88, 9.52)	11.5 (9.34, 14.08)	7.6 (5.97, 9.67)
		**AARC**	15.17	1.04	−1.62	7.57	12.1
Regional	Non-Indigenous	NHS 2014–2015	6.8 (5.98, 7.84)	47.6 (45.54, 49.68)	6.2 (5.33, 7.19)	18.4 (16.9, 19.93)	10.0 (8.96, 11.2)
		NHS 2017–2018	5.4 (4.66, 6.19)	47.5 (45.66, 49.35)	5.7 (4.96, 6.49)	16.8 (15.51, 18.13)	8.7 (7.76, 9.67)
		**AARC**	−7.79	−0.07	−2.88	−2.97	−4.71
	Indigenous	NATSIHS 2012–2013	3.8 (2.73, 5.29)	58.4 (54.81, 61.97)	10.4 (8.47, 12.64)	7.4 (5.88, 9.36)	4.2 (3.1, 5.65)
		NATSIHS 2018–2019	7.9 (6.32, 9.75)	59.7 (56.26, 62.98)	10.6 (8.95, 12.59)	15.0 (12.84, 17.54)	8.0 (6.39, 9.91)
		**AARC**	12.85	0.35	0.41	12.47	11.32
Inner regional	Non-Indigenous	NHS 2014–2015	7.1 (6.02, 8.44)	46.4 (43.86, 49.02)	6.0 (4.99, 7.21)	19.7 (17.81, 21.73)	10.2 (8.9, 11.77)
		NHS 2017–2018	5.3 (4.39, 6.28)	47.2 (44.94, 49.51)	5.2 (4.41, 6.22)	17.1 (15.57, 18.85)	9.0 (7.89, 10.31)
		**AARC**	−9.69	0.56	−4.41	−4.51	−4.13
	Indigenous	NATSIHS 2012–2013	3.3 (2.04, 5.18)	57.8 (52.83, 62.54)	9.5 (7.17, 12.4)	6.8 (4.95, 9.34)	4.5 (2.99, 6.61)
		NATSIHS 2018–2019	8.1 (6.24, 10.41)	61.1 (56.4, 65.63)	10.6 (8.39, 13.44)	15.0 (12.18, 18.26)	7.5 (5.67, 9.78)
		**AARC**	16.33	0.94	1.98	14	8.98
Outer regional	Non-Indigenous	NHS 2014–2015	6.3 (5.01, 7.85)	50.0 (46.49, 53.38)	6.6 (5.06, 8.46)	15.7 (13.58, 18.15)	9.6 (7.96, 11.48)
		NHS 2017–2018	5.6 (4.49, 7.07)	48.1 (45.08, 51.22)	6.65 (5.36, 8.23)	15.9 (13.92, 18.16)	7.9 (6.5, 9.46)
		**AARC**	−3.52	−1.2	0.45	0.42	−6.35
	Indigenous	NATSIHS 2012–2013	4.4 (2.77, 7.03)	59.2 (53.84, 64.38)	11.4 (8.49, 15.18)	8.1 (5.77, 11.34)	3.9 (2.44, 6.11)
		NATSIHS 2018–2019	7.6 (5.26, 10.92)	58.0 (53.03, 62.79)	10.6 (8.26, 13.53)	15.1 (11.82, 19.14)	8.5 (6.03, 11.99)
		**AARC**	9.42	−0.35	−1.2	10.89	14.08
Remote	Non-Indigenous	NHS 2014–2015	4.5 (2.27, 8.92)	41.3 (33.64, 49.48)	6.4 (3.4, 11.71)	14.3 (9.58, 20.87)	8.1 (4.41, 14.57)
		NHS 2017–2018	7.1 (3.86, 12.56)	48.5 (41.27, 55.73)	7.5 (4.32, 12.87)	13.1 (9.31, 18.26)	6.4 (3.49, 11.38)
		**AARC**	15.77	5.44	5.72	−2.8	−7.8
	Indigenous	NATSIHS 2012–2013	7.9 (6.87, 9.2)	55.5 (53.18, 57.86)	11.9 (10.59, 13.37)	17.0 (15.44, 18.76)	7.0 (6.03, 8.09)
		NATSIHS 2018–2019	7.8 (6.47, 9.37)	56.5 (53.69, 59.25)	13.0 (11.26, 14.99)	16.0 (14.15, 18.05)	6.5 (5.46, 7.78)
		**AARC**	−0.34	0.29	1.48	−1.02	−1.12

*NHS* national health survey, *NATSIHS* National Aboriginal and Torres Strait Islander Health Survey, *CI* confidence interval, *AARC* average annual rate of change (negative and positive signs represent a decrease and an increase in the prevalence, respectively).

^a^Weighted estimates.

Central obesity exhibited a geographic distribution that was similar to overall MetS, with a higher prevalence observed in rural and remote areas. Among non-Indigenous adults, central obesity prevalence in 2017–2018 was highest in Remote areas at 48.5% (95% CI: 41.3–55.7) and lowest in Major Cities at 38.4% (37.3–39.6). Indigenous adults in 2018–2019 had the highest central obesity prevalence in Inner Regional areas (61.1%, 95% CI: 56.4–65.6), with the lowest in Major Cities (55.3%, 95% CI: 51.0–59.6) (Table 3). Notably, even in urban settings, more than half of Indigenous adults were affected by central obesity. Other MetS risk factors showed a similar urban–rural gradient, with higher prevalences reported outside of Major Cities for both populations. For example, T2D and hypertension were more common in Regional and Remote areas than in Major Cities (Table 3). An exception to the overall pattern was observed for hypercholesterolaemia, which showed lower prevalence in Remote areas than in Major Cities. In Remote regions, high cholesterol affected 6.5% (95% CI: 5.5–7.8) of Indigenous and 6.4% (95% CI: 3.5–11.4) of non-Indigenous adults, compared to 7.6% of those in Major Cities (Indigenous: 95% CI: 5.97–9.67; non-Indigenous: 95% CI: 7.02–8.19) (Table 3).

### Geographic trends in MetS and its risk factors by Indigenous status

The prevalence of MetS demonstrated contrasting temporal trends by Indigenous status and geographic distribution. Among non-Indigenous adults, the prevalence of MetS declined from 5.5% in 2014–2015 to 4.6% in 2017–2018 (AARC: –6.07%), despite a slight increase in central obesity during the same period. This overall decline was primarily driven by modest reductions in T2D, hypertension, and particularly high cholesterol (Table 2). In contrast, MetS prevalence increased among Indigenous adults, rising from 4.1% in 2012–2013 to 7.1% in 2018–2019 (AARC: +9.59%) (Table 2). Central obesity remained the most prevalent MetS risk factor in both populations, increasing slightly from 39.9% to 40.9% among non-Indigenous adults (AARC: +0.78%) and from 55% to 57.4% among Indigenous adults (AARC: +0.71%) (Table 2). The prevalence of T2D remained relatively stable over the study period among both Indigenous adults (9.8% to 9.9%) and non-Indigenous adults (5.0% to 4.8%) (Table 2). While there was a slight numerical decrease among non-Indigenous adults, the magnitude of change in both groups was minimal and unlikely to be clinically significant. Hypertension increased notably in Indigenous adults (9.5% to 13.9%; AARC: +6.5%) but declined modestly in non-Indigenous adults (14.6% to 13.7%; AARC: –2.06%). High cholesterol declined among non-Indigenous adults (9.1% to 7.8%; AARC: –4.98%) but rose markedly among Indigenous adults (4.7% to 7.6%; AARC: +8.17%) (Table 2).

**Fig. 1:**
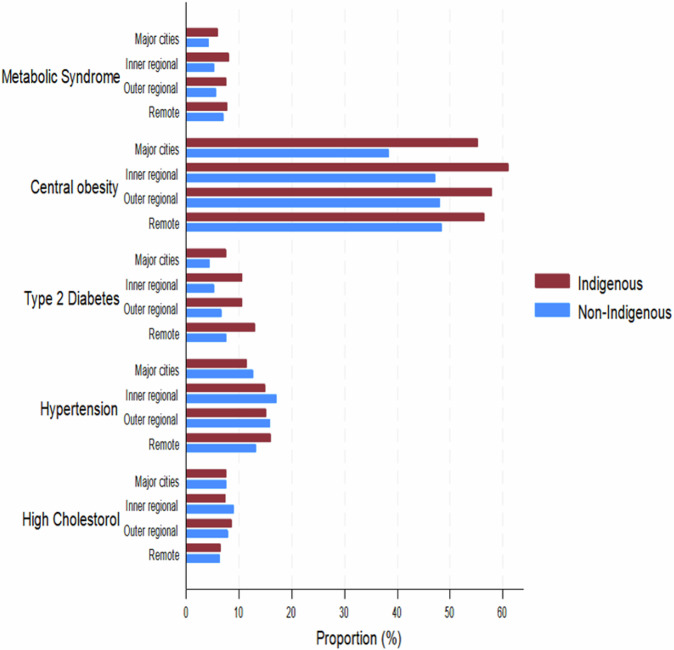
Prevalence of metabolic syndrome and its major risk factors by remoteness for Indigenous and non-Indigenous Australians. Prevalence estimates of metabolic syndrome, central obesity, type 2 diabetes, hypertension, and high cholesterol among Indigenous and non-Indigenous Australian adults across Major Cities, Inner Regional, Outer Regional, and Remote areas, based on data from the National Health Survey (2014–2015 and 2017–2018) and the National Aboriginal and Torres Strait Islander Health Survey (2012–2013 and 2018–2019).

MetS prevalence increased with remoteness and showed widening disparities across population groups. In 2018–2019, prevalence was higher in Remote areas than in Major Cities for both Indigenous (7.8% vs. 5.9%) and non-Indigenous (7.1% vs. 4.3%) adults (Table 3). The most contrasting trends were observed in Inner Regional areas: Indigenous prevalence rose from 3.3% to 8.1% (AARC: +16.33%), while non-Indigenous prevalence declined from 7.1% to 5.3% (AARC: –9.69%) (Table 3). In Major Cities, Indigenous prevalence more than doubled (2.5% to 5.9%), while non-Indigenous prevalence declined modestly (5.1% to 4.3%). In Remote areas, MetS prevalence rose substantially among non-Indigenous adults (4.5% to 7.1%; AARC: +15.77%) but remained stable in Indigenous adults (7.9% to 7.8%; AARC: –0.34%) (Table 3). In the most recent surveys, Indigenous adults exhibited higher MetS prevalence than non-Indigenous adults in all geographic regions, with the largest disparities observed in regional areas (Fig. 1).

Major MetS risk factors varied across regions and populations. Central obesity increased slightly in most areas, with Indigenous adults consistently bearing the highest burden. Among non-Indigenous adults, the largest increase occurred in Remote areas (41.3% to 48.5%; AARC: +5.44%), while the most notable rise among Indigenous adults was observed in Major Cities (52.0% to 55.3%; AARC: +1.04%) (Table 3). The prevalence of T2D declined in non-Indigenous adults across most regions, especially in Inner Regional areas (6.0% to 5.2%; AARC: –4.41%) but increased in Remote areas (6.4% to 7.5%; AARC: +5.72%). Among Indigenous adults, T2D prevalence rose in Inner Regional (9.5% to 10.6%; AARC: +1.98%) and Remote areas (11.9% to 13.0%; AARC: +1.48%), while declining slightly in Major Cities (Table 3). Hypertension declined across all regions among non-Indigenous adults, with the largest reduction observed in Inner Regional areas (19.7% to 17.1%; AARC: –4.51%). In contrast, hypertension prevalence increased considerably among Indigenous adults, particularly in Inner Regional areas (6.8% to 15.0%; AARC: +14.0%). A slight decrease was observed in Remote areas among Indigenous adults, where hypertension fell from 17% to 16% (AARC: –1.02%) (Table 3). High cholesterol also declined in all regions among non-Indigenous adults, with the most pronounced drop in Remote areas (8.1% to 6.4%; AARC: –7.8%). Conversely, Indigenous adults experienced increases in high cholesterol across all areas except Remote areas, with the sharpest rise in Outer Regional areas (3.9% to 8.5%; AARC: +14.08%). Notably, remote areas had the lowest cholesterol prevalence for both populations by the latest survey years, contrasting with the distribution of other MetS risk factors (Fig. 1).

## Discussion

This study presents the first nationally representative analysis of MetS prevalence, major risk factors, trends and geographic variation among Indigenous and non-Indigenous Australians. By integrating data from the NHS and NATSIHS across multiple time points, we found significant disparities in MetS prevalence and its associated risk factors, particularly by ethnicity and remoteness. Indigenous Australians consistently exhibited a higher burden of MetS and its components, especially central obesity and self-reported T2D, compared to non-Indigenous Australians. Between 2012–2013 and 2018–2019, MetS prevalence increased among Indigenous adults, whereas non-Indigenous adults experienced a modest decline, a divergence of particular concern given the established association between MetS and elevated risk of CVD, T2D, macular degeneration, and CKD [6, 9, 45]. These findings are consistent with national reports documenting persistently higher rates of chronic disease among Indigenous Australians and in rural and remote communities [46–48]. Central obesity emerged as the most prevalent MetS component in both populations, affecting more than half of Indigenous adults and exceeding the global average of 41% [49, 50]. This disproportionate burden represents a significant public health threat for Indigenous communities.

Central abdominal obesity is a critical driver of insulin resistance and cardiometabolic disease [51]. Waist circumference, as used in this study, is a stronger indicator of visceral adiposity and metabolic risk than body mass index (BMI) [52]. The disproportionately high prevalence among Indigenous adults reflects longstanding inequalities in nutrition, physical activity, and access to preventive healthcare [47, 53]. Beyond individual health behaviours, historical trauma, intergenerational disadvantage, and colonisation-related disruption of cultural practices have also contributed to this burden. A shift from traditional food systems to energy-dense, nutrient-poor products, reinforced by high food prices and constrained access to affordable fresh produce in many remote and regional communities, has further driven central adiposity [48, 54–56]. Overcrowded and insecure housing, inadequate access to safe spaces for physical activity, and chronic psychosocial stress reduce opportunities for healthy living and are associated with greater waist circumference and elevated cardiometabolic risk [57, 58]. Given the central role of abdominal obesity in the pathophysiology of MetS, these findings emphasise the urgent need for targeted, culturally safe interventions in Indigenous communities to reduce this burden.

T2D prevalence was substantially higher among Indigenous adults and remained relatively stable over time, in contrast to the slight decline observed among non-Indigenous adults. This persistent disparity reflects the well-documented, disproportionate burden of diabetes among Indigenous Australians [18–21]. The highest prevalence was observed in remote Indigenous communities, reaching 13%, which is nearly double the prevalence reported in major cities. This geographic gradient reflects broader patterns in the distribution of chronic disease and is shaped by structural inequities such as limited health care access, food insecurity, and substandard living conditions [47, 59]. Addressing these entrenched challenges requires community-led, locally tailored interventions that target both behavioural risk factors and the social determinants of health.

Geographic disparities in MetS prevalence were most pronounced in remote communities. While non-Indigenous adults experienced improvements across most remoteness categories, prevalence increased notably in remote areas. In contrast, Indigenous adults exhibited a substantial rise in inner regional and urban areas, while prevalence remained relatively stable in remote regions. These patterns differ from global trends, where urban populations typically show higher MetS prevalence due to greater exposure to sedentary lifestyles and processed foods [60]. In the Australian context, this inverse gradient likely reflects persistent socio-structural inequalities, including limited access to health care, food insecurity, and suboptimal dietary practices in remote communities [18]. These challenges are particularly pronounced among Indigenous populations [47, 59, 61]. The steeper increase among Indigenous adults in inner regional areas may reflect rapid nutrition and lifestyle transitions, the influence of obesogenic retail food environments, and less consistent access to culturally safe preventive care compared with that available in major cities [54, 62, 63]. Greater uptake of Indigenous-specific Medicare-funded health checks in regional settings relative to very remote areas, may also have contributed to higher detection of metabolic abnormalities [64]. In very remote communities, where obesity and T2D are already highly prevalent, there may be limited scope for further measurable increases [47].

Culturally appropriate, community-led nutritional interventions are urgently needed to address the growing burden of MetS among Indigenous Australians and those living outside major cities, particularly in rural and remote areas. Programmes promoting healthy diets, physical activity, and routine health screening should be co-designed and implemented with Aboriginal Community Controlled Health Organisations (ACCHOs) and Local Health Districts (LHDs), which are well-positioned to deliver culturally safe and effective care [65, 66]. Evidence shows that interventions grounded in traditional knowledge such as incorporating bush foods, community gardens, and Indigenous games, can enhance engagement and promote sustained behaviour change [54]. In regional and remote non-Indigenous populations, where MetS and its components have risen, tailored prevention is equally important. These communities may be underserved by urban-centric prevention strategies. Expanding rural health services, scaling up telehealth, and investing in region-specific health promotion could help reverse these trends. Addressing broader social determinants, such as poverty, housing insecurity, and limited educational opportunities, is fundamental. A multisectoral approach that integrates health, education, agriculture, and housing sectors is required to overcome structural barriers to healthy living for both Indigenous and non-Indigenous Australians [48, 67].

Geographic variation in MetS prevalence calls for regionally tailored policy response. In major cities, where prevalence is lower but rising among Indigenous adults, priorities include weight management, culturally appropriate nutritional support, and improved preventive screening through ACCHOs. In inner regional areas, where the sharpest increases were observed, strengthening chronic disease programmes and expanding culturally safe primary care are essential. In outer regional areas, where service availability is constrained, workforce retention, telehealth expansion, and school and community-based health promotion should be emphasised. In remote areas, poor outcomes are compounded by workforce instability, high food costs, and inadequate infrastructure [68, 69]. Policy strategies should therefore extend beyond food supply and housing to ensure local access to core primary health care, even in very small communities. Evidence indicates that chronic and metabolic disease management is most effective when delivered by resident primary health care providers, even in smaller communities, and guided by principles of equity, cultural safety, and accessibility of care [48, 68, 69].

Between 2012 and 2019, MetS prevalence increased among Indigenous Australians but declined modestly among non-Indigenous adults, reflecting the combined influence of structural determinants, behavioural risk factors, and health system changes. Among Indigenous populations, overweight and obesity rose from 66% in 2012–2013 to 74% in 2018–2019 among adults, suggesting a growing burden of metabolic risk during this period, although causality cannot be inferred due to the cross-sectional design [70, 71]. This trend occurred within a context of entrenched socioeconomic disadvantage, food insecurity, poor availability and affordability of nutritious foods, and systemic inequities recognised as key determinants of obesity and T2D [48]. During the same period, uptake of Medicare funded annual health assessments for Indigenous Australians expanded, improving detection of hypertension, diabetes, and dyslipidaemia and likely contributing to the higher recorded prevalence of MetS [64]. In contrast, the modest decline observed among non-Indigenous adults aligns with improved risk factor management, including reductions in self-reported high cholesterol from 7.1% in 2014–2015 to 6.1% in 2017–2018, supported by sustained dispensing of statins and other lipid-lowering medicines between 2013 and 2019 [72, 73]. Overall, these findings suggest that rising obesity, persistent social disadvantage, and enhanced case detection are intensifying metabolic risk among Indigenous Australians, while improved clinical management has stabilised prevalence among non-Indigenous adults, underscoring the need to reduce inequities while sustaining progress in the broader population.

### Strengths and limitations

This study leveraged large, nationally representative datasets (NHS and NATSIHS) and conducted stratified analyses by remoteness and ethnicity, providing valuable insights into geographic and population-based disparities in MetS. The use of consistent data collection methods across survey years enhances comparability, and the application of population weights and the calculation of average annual rate of change (AARC) add rigour to trend analysis.

However, several limitations should be acknowledged when interpreting these findings. First, the reliance on self-reported data for conditions such as diabetes (elevated blood glucose), high cholesterol, and partly for blood pressure may have introduced misclassification. These conditions are prone to both underestimation and overestimation due to recall error and social desirability bias, with self-reported blood pressure particularly vulnerable. These reporting and measurement limitations may affect estimates for individual conditions and, in turn, contribute to misclassification of MetS status, because classification requires multiple risk factors. National estimates suggest that up to 30% of people living with diabetes in Australia remain undiagnosed, underscoring that self-reported survey data may not fully capture the true burden of disease, particularly in underserved populations [74]. Second, although survey weights accounted for the complex sampling design and unequal selection probabilities by remoteness, the survey selected up to two adults per household in non-remote areas and one adult in remote areas. This differential within-household sampling may introduce systematic bias, particularly if multiple individuals in remote households share similar cardiometabolic risk profiles. Such design features could result in underestimation of MetS prevalence in remote populations, especially where risk factors are known to cluster. Third, the cross-sectional design of the NHS and NATSIHS precludes causal inference and limits the ability to observe individual level changes over time. Fourth, the exclusion of very remote communities from the NHS sample may have led to an underrepresentation of populations experiencing the most significant health disparities. Finally, our operational definition excluded HDL cholesterol and triglycerides because these biomarkers were not consistently available across survey waves. Standard definitions (ATP III, IDF, and the Joint Interim Statement) define dyslipidaemia as low HDL cholesterol and elevated triglycerides [6, 7, 10, 39], whereas our reliance on a general ‘high cholesterol’ indicator did not fully capture this profile. As a result, some individuals who would qualify under standard criteria were likely missed, making our prevalence estimates conservative and limiting comparability with studies using full lipid panels [10, 39]. Despite these limitations, the use of large, nationally representative datasets, together with a consistent methodology across survey waves, strengthens the validity of the findings and supports their relevance for guiding public health policy and targeted intervention strategies.

## Conclusion

This study highlights significant disparities in MetS prevalence across Australia, with Indigenous Australians and residents of regional and remote areas bearing the greatest burden. Among non-Indigenous adults, prevalence declined overall but rose in remote areas. Among Indigenous adults, prevalence increased in Major Cities and in inner and outer regional areas, whereas it remained stable in remote communities. Central obesity and T2D were the principal contributors to MetS among Indigenous adults, whereas hypertension and high cholesterol were more common among non-Indigenous adults in regional areas. These findings highlight the urgent need for culturally responsive and geographically targeted public health strategies. Priorities include strengthening partnerships with LHD and ACCHOs, improving access to affordable nutritious food, promoting physical activity, and expanding preventive care with consistent follow-up after Medicare-funded Indigenous health checks, alongside stronger continuity of care between urban, regional, and remote services. Future surveillance should incorporate lipid biomarkers and include very remote communities, guided by Indigenous data governance, to enable accurate monitoring. Reducing the burden of MetS in Australia requires coordinated public health action that addresses central obesity, T2D, and hypertension, and delivers region-specific prevention and culturally safe primary care across urban, regional, and remote settings for both Indigenous and non-Indigenous populations.

## Data Availability

This study used data from two surveys conducted by the Australian Bureau of Statistics (ABS): the National Health Survey (NHS) and the National Aboriginal and Torres Strait Islander Health Survey (NATSIHS). Weighted estimates from both surveys are available on the ABS website. Access to the de-identified unit record microdata is provided through the secure ABS DataLab to approved users, and all outputs are reviewed by ABS prior to release.
